# ^225^Ac-PSMA-617 in chemotherapy-naive patients with advanced prostate cancer: a pilot study

**DOI:** 10.1007/s00259-018-4167-0

**Published:** 2018-09-19

**Authors:** Mike Sathekge, Frank Bruchertseifer, Otto Knoesen, Florette Reyneke, Ismaheel Lawal, Thabo Lengana, Cindy Davis, Johncy Mahapane, Ceceila Corbett, Mariza Vorster, Alfred Morgenstern

**Affiliations:** 1 Department of Nuclear Medicine, Steve Biko Academic Hospital, University of Pretoria, Private Bag X169, Pretoria, 0001 South Africa; 2 European Commission, Joint Research Centre, Directorate for Nuclear Safety and Security, Karlsruhe, Germany; 3 Nuclear Technology Products (NTP), Pelindaba, South Africa

**Keywords:** Prostate cancer, Actinium-225, PSMA, Radioligand therapy, Chemotherapy-naïve, PSA response

## Abstract

**Background:**

A remarkable therapeutic efficacy has been demonstrated with ^225^Ac-prostate-specific membrane antigen (PSMA)-617 in heavily pre-treated metastatic castration-resistant prostate cancer (mCRPC) patients. We report our experience with ^225^Ac-PSMA-617 therapy in chemotherapy-naïve patients with advanced metastatic prostate carcinoma.

**Methods:**

Seventeen patients with advanced prostate cancer were selected for treatment with ^225^Ac-PSMA-617 in 2-month intervals, with initial activity of 8 MBq, then de-escalation to 7 MBq, 6 MBq or 4 MBq in cases of good response. In one patient, activity was escalated to 13 MBq in the third cycle. Fourteen patients had three treatment cycles administered, while in three patients treatment was discontinued after two cycles due to good response. Six out of 17 patients received additional treatments after the third cycle. Prostate-specific antigen (PSA) was measured every 4 weeks for PSA response assessment. ^68^Ga-PSMA-PET/CT was used for functional response assessment before each subsequent treatment cycle. Serial full blood count, renal function test, and liver function were obtained to determine treatment-related side effects.

**Results:**

Good antitumor activity assessed by serum PSA level and ^68^Ga-PSMA-PET/CT was seen in 16/17 patients. In 14/17 patients, PSA decline ≥90% was seen after treatment, including seven patients with undetectable serum PSA following two (2/7) or three cycles (5/7) cycles of ^225^Ac-PSMA-617. Fifteen of 17 patients had a > 50% decline in lesions avidity for tracer on ^68^Ga-PSMA-PET/CT including 11 patients with complete resolution (PET-negative and either stable sclerosis on CT for bone or resolution of lymph node metastases) of all metastatic lesions. Grade 1/2 xerostomia was seen in all patients, and none was severe enough to lead to discontinuation of treatment. One patient had with extensive bone marrow metastases and a background anemia developed a grade 3 anemia while another patient with solitary kidney and pre-treatment grade 3 renal failure developed grade 4 renal toxicity following treatment. The group presented with significant palliation of bone pain and reduced toxicity to salivary glands due to de-escalation.

**Conclusions:**

^225^Ac-PSMA-617 RLT of chemotherapy-naïve patients with advanced metastatic prostate carcinoma led to a ≥ 90% decline in serum PSA in 82% of patients including 41% of patients with undetectable serum PSA who remained in remission 12 months after therapy. The remarkable therapeutic efficacy reported in this study could be achieved with reduced toxicity to salivary glands due to de-escalation of administered activities in subsequent treatment cycles. This necessitates further exploration for informing clinical practice and clinical trial design.

## Introduction

Prostate cancer (PC) is one of the most common cancer types and a leading cause of cancer-related death in men [1]. The 5-year survival rate of localized prostate cancer is excellent; however, metastatic disease is associated with poorer outcome [2]. Wide variations exist in the mortality due to prostate cancer across different regions of the world. Late presentation when the disease is already widely metastatic, limited availability of the effective newer treatment agents and genetic differences are some of the putative factors responsible for this disparity in mortality [2–5].

Androgen deprivation therapy is the standard of care for advanced or metastatic prostate cancer in patients with hormone-sensitive disease. Initially, almost all patients with hormone-naive PC have a good response to the well-established anti-androgen treatments [6]. However, in high Gleason score patients, resistance to these treatments occurs frequently within 1 to 2 years. Advanced prostate cancer, over time, also become refractory to the newly expanded treatment options such as cytotoxic agents, Radium-223, immunotherapy, and bone supportive agents [7, 8]. Additionally, due to potential toxicities, chemotherapeutic agents may not be suitable for all patients [9].

There are no validated clinical decision tools to aid optimal treatment selection in men with metastatic castration-resistant prostate cancer (mCRPC). Frequently, abiraterone and enzalutamide are used prior to chemotherapy in mCRPC, given the more favorable safety profiles. Currently, the decision of how to best use these therapies is based on anecdotal evidence, physician and patient preferences, cost considerations, and side effect profiles [10].

Prostate-specific membrane antigen (PSMA) is overexpressed in metastatic prostate cancer and has been found to be a suitable target for imaging and therapy [11–13]. The PSMA ligand PSMA-617 is a highly promising novel compound for therapy of prostate cancer [14].

The ^225^Actinium labeled derivative, ^225^Ac-PSMA-617, developed and characterized by the JRC Karlsruhe in 2013, has shown a remarkable therapeutic efficacy in heavily pre-treated metastatic castration-resistant prostate cancer (mCRPC) patients [15–17]. PSMA-based radioligand therapy (RLT) approach could be an attractive therapy option for advanced prostate cancer. Here we report our initial experience with ^225^Ac-PSMA-617 RLT in the first 17 consecutive, chemotherapy-naïve patients with advanced metastatic prostate cancer treated at Steve Biko Academic Hospital, in Pretoria, South Africa. For all patients reported here at least 1 year follow up is available after initiation of therapy (median 13 ± 2 months).

## Patients and methods

This is a retrospective review of chemotherapy-naïve patients with histologically confirmed metastatic prostate cancer treated with ^225^Ac-PSMA-617 RLT. The treatment group consists of patients who relapsed after initial therapy with radical prostatectomy, bilateral orchidectomy, external beam radiotherapy, prostate brachytherapy and androgen deprivation therapy with gonadotropin-releasing hormone analogs or treatment-naïve patients who presented with widespread metastatic prostate carcinoma. Inclusion criteria included Eastern Cooperative Oncology Group (ECOG) performance status score 2 or lower, a life expectancy of 6 months or more, widespread metastatic disease precluding treating with radiotherapy, patients’ refusal of chemotherapy or hormonal therapy, and lack of access to second-generation anti-androgen therapy (abiraterone and enzalutamide). These novel anti-androgen medications are not available in the state-owned hospitals in South Africa and are only accessible to patients with medical insurance. We excluded patients with impaired bone marrow function (hemoglobin concentration < 8 g/dL, platelet counts <75 × 10^9^/L, or white blood cell count <3.0 × 10^9^/L), compromised renal function defined as glomerular filtration rate of <30 mL/min/1.73m^2^ body surface area or impaired liver function defined as albumin <25 g/L. The decision to treat patients with ^225^Ac-PSMA-617 RLT was made by the local interdisciplinary tumor board. All patients were aware that ^225^Ac-PSMA-617 is an experimental treatment agent not approved either in South Africa or elsewhere in the world. We informed patients of possible adverse events related to this treatment especially dry mouth, bone marrow suppression, renal impairment and a possibility of any other side effects that may be unknown at this time. Treatments were administered only after patients gave written informed consent.

### Patient preparation

All patients underwent ^68^Ga-PSMA-11 PET/CT imaging as part of an initial assessment to determine suitability for ^225^Ac-PSMA-617 treatment. Patients were considered suitable for treatment if metastatic lesions identified demonstrated sufficient expression of the PSMA defined as uptake greater than twice the physiologic uptake in the normal liver. Synthesis of ^68^Ga-PSMA-11 and the performance of PET/CT imaging were as we have previously reported [18, 19].

Within 2 weeks of scheduled treatment with ^225^Ac-PSMA-617, patients had the following investigations: full blood count; liver function tests; electrolytes, urea, and creatinine; and GFR. These investigations were repeated 2 weeks before subsequent treatment cycles to determine early toxicity and suitability for more treatment cycles. Dynamic renal scintigraphy using ^99m^Tc-MAG3 was performed at baseline in all patients to rule out urinary tract obstruction.

### Preparation and administration of ^225^Ac-PSMA-617

The PSMA-617 precursor (ABX advanced biochemical compounds, Radeberg, Germany) was labeled with ^225^Ac (JRC, Karlsruhe, Germany) in-house and administered to patients as previously reported [15]. The initial administered activity was 8 MBq. Administered activity was de-escalated in subsequent treatment cycles to 7, 6 or 4 MBq based on response to earlier administered treatment. Treatment was repeated after every 8 weeks.

### Evaluation of response and toxicity

For all patients reported here at least 1 year follow up is available after initiation of therapy (median 13 ± 2 months). We determined response to treatment using serial measurements of serum PSA and ^68^Ga-PSMA-PET/CT imaging. PSA was obtained at baseline and subsequently every 4 weeks to determine PSA response to therapy. ^68^Ga-PSMA-PET/CT was repeated every 8 weeks (before each subsequent cycle of treatments was administered) and every 12 weeks after completion of treatment until disease progression. PSA response rate was evaluated using the Prostate Cancer Working Group 3 (PCWG3) criteria defined as a PSA decline ≥50% as a biochemical response [20]. Additionally, any change in PSA values was documented and analyzed. Follow-up ^68^Ga-PSMA-11 PET/CT was used to define resolution of initially identified metastatic lesions on baseline scan.

We followed-up patients with serial investigations including full blood count; liver function tests; electrolytes, urea, and creatinine; and GFR every 12 weeks after completion of treatment cycles to determine post-treatment toxicity. We defined toxicity according to the common terminology criteria for adverse events version 5.0 (CTCAE v5.0). Premised on previous studies of PSMA-based radionuclide therapy reporting xerostomia as a common side effect, we specifically questioned each patient regarding this side effect. Xerostomia was also defined using the CTCAE v5.0 which grades it from 1 to 3 with grade 1 toxicity being symptomatic dry mouth without significant dietary alteration while grade 3 toxicity is defined as an inability to tolerate oral feeding requiring total parenteral feeding or tube feeding.

### Statistical analysis

Descriptive statistics as absolute and relative frequencies, mean and standard deviation were used to characterize the study population. PSA response was defined as a maximum change in PSA that amounts to a decline ≥50%. The change in PSA is illustrated by waterfall plots that show the PSA change of individual patients and are sorted by the extent of change. We compared pre-treatment and follow-up leucocyte count, hemoglobin level, platelet count, serum creatinine, and serum albumin levels using paired t-Test. Statistical analysis was done using MedCalc®. Statistical significance was set at a *p* value of <0.05.

## Results

A total of 59 cycles of ^225^Ac-PSMA-617 RLT were administered to 17 men, mean age = 64.5 ± 9.7 years (range: 45 to 82). The mean administered activity was 7.4 ± 1.5 MBq. Fourteen of 17 patients received the scheduled three cycles of ^225^Ac-PSMA-617 RLT. In 3/17 patients, treatment was discontinued after two cycles due to excellent response. These patients with excellent response after two cycles of treatment had their serum PSA levels declined to below detectable limits and on the ^68^Ga-PSMA-11 PET/CT scan done for re-staging, metastatic lymph nodes were less than 1 cm in diameter with no residual avidity for tracer. Six out of 17 patients received additional treatments after the third cycle (median one additional cycle). In 13 out of 17 patients the administered activity of ^225^Ac-PSMA-617 could be de-escalated from 8 MBq in the first treatment cycle to activities between 7 and 4 MBq in subsequent cycles due to favorable responses. The majority of patients had an ECOG performance of 0 (13) while two patients each had a score of 1 and 2. Table 1 shows the baseline characteristics of the study population.

**Table 1:** Baseline characteristics of the treatment group

Patient No.	Age (years)	PSA level	Gleason score	Prior treatment	Sites of metastases	ECOG performance status	No. of cycles ^225^Ac-PSMA-617	Activity administered per cycle (MBq)
1	78	33.84	9(4 + 5)	Radical prostatectomy, EBRT, ADT	Bones and lymph nodes, liver, brain	0	3	8.1/ 7.9/ 7.3
2	58	102.82	9(4 + 5)	Brachytherapy, EBRT, ADT	Bones	2	4	7.9/ 8.6/ 8.0/ 7.0
3	66	204.70	9(4 + 5)	Brachytherapy, EBRT, ADT, 4 cycles of ^177^Lu-177 PSMA	Bones	0	6	8.2/ 8.0/ 13.4/ 8.0/ 6.2/4.1
4	60	49.08	6(3 + 3)	Brachytherapy, ADT	Bones and lymph nodes	1	5	8.6/ 8.3/ 7.2/ 6.2/ 6.0
5	66	6.28	7(4 + 3)	Radical prostatectomy, ADT, EBRT	Bones	0	4	8.4/ 8.4/ 6.1/ 4.5
6	75	94.67	8(4 + 4)	Radical prostatectomy, EBRT, ADT, 6 cycles of ^177^Lu-PSMA	Bones	1	2	8.0/ 7.3
7	58	1.20	7(3 + 4)	Bilateral orchidectomy, ADT	Lymph nodes	0	2	8.3/ 4.0
8	75	14.04	7(4 + 3)	None	Bones	0	3	8.6/ 5.9/ 6.2
9	66	71.41	8(4 + 4)	None	Bones, lymph nodes, and lung	0	4	8.3/ 8.1/ 7.2/ 6.6
10	55	6.70	9(4 + 5)	ADT	Bones and lymph nodes	0	3	8.3/ 7.5/ 7.0
11	62	9.33	9(4 + 5)	ADT	Lymph nodes	0	2	8.2/ 4.0
12	45	15.33	9(4 + 5)	ADT	Bones and lymph nodes	2	3	8.3/ 8.2/ 7.9
13	82	8.03	7(4 + 3)	Radical prostatectomy, EBRT, ADT	Bones	0	3	8.0/ 6.3/ 6.1
14	69	100.87	6(3 + 3)	Bilateral orchidectomy	Bones and lymph nodes	0	3	8.6/6.3/ 6.4
15	65	212.90	9(4 + 5)	None	Bones	0	3	8.4/ 8.1/ 7.5
16	66	782.00	9(4 + 5)	None	Bones	0	3	8.5/ 7.3/ 6.2
17	51	1300.69	10(5 + 5)	1 cycle of ^177^Lu-PSMA	Lymph nodes	0	3	8.0/6.3/ 4.5

ECOG: Eastern Cooperative Oncology Group; ADT: Androgen Deprivation Therapy; PSA: Prostate Specific Antigen; EBRT: External Beam Radiotherapy; ^177^Lu-PSMA: Lutetium 177-labeled Prostate-Specific Membrane Antigen

All patients included demonstrated widely metastatic prostate carcinoma with 10 or more metastatic foci seen in each patient. Bone metastases were seen in 82% of patients while 50% of patients had metastases to the lymph nodes. Of the 17 patients treated, eight patients had bone-only metastases, three patients had lymph node-only metastases, and six had a combination of lymph node and bone metastases. Two patients had visceral metastases; one with lung metastases and the other with brain and liver metastases. These latter two patients had visceral metastases in addition to lymph node and skeletal metastases.

### Response

**Fig. 1:**
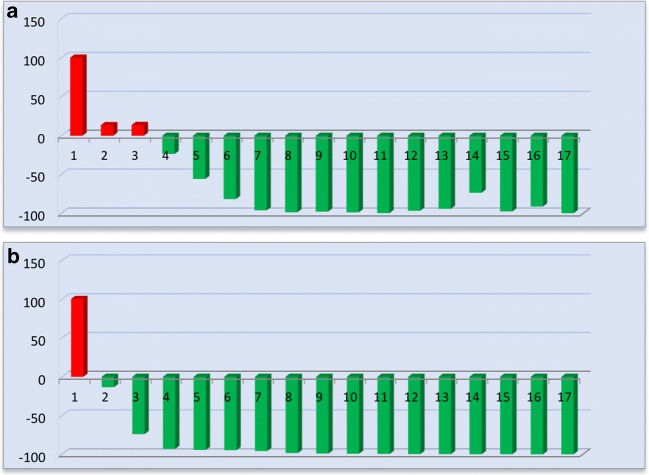
**a** Waterfall plot showing PSA response at 8 weeks after one cycle of ^225^Ac-PSMA-617 radioligand therapy. **b** Waterfall plot showing best PSA response to ^225^Ac-PSMA-617 radioligand therapy

Following two (2/7) or three (5/7) cycles of ^225^Ac-PSMA-617, seven patients had undetectable serum PSA (<0.1 ng/mL). We found a PSA decline ≥90% in 14 of 17 patients at the completion of treatment. After the first cycle of treatment, a PSA decline of ≥80% was seen in 12/17 patients, decline of <80%, but ≥50% in 1/17 patients and a decline of <50% in 1/17 patient (Fig. 1a). An initial rise in PSA level was seen in 3/17 patients; one of whom eventually had a PSA decline of 74% at the end of treatment (achieved a best PSA decline to 53.3 ng/mL from a baseline of 204.7 ng/mL), Fig. 1b. Another patient with an initial rise in PSA showed a further rise in PSA level after the second and third cycle of and treatment with ^225^Ac-PSMA-617 was subsequently discontinued.

Restaging based on the ^68^Ga-PSMA-11 PET/CT imaging showed a > 50% reduction in tracer avidity of metastatic nodal and skeletal lesions in 15/17 patients. Response on PET scan was seen as reduction in tracer avidity in metastatic lesions, as well as reduction in the size of metastatic lymph nodes or visceral metastases and stable sclerosis at the sites of bone metastases. Two patients showed appearance of new lesions on follow-up ^68^Ga-PSMA-11 PET/CT compared with baseline scan. In 11 patients, all lesions had resolved entirely after two or three cycles of treatment with tracer uptake at the sites of previously identified metastases comparable to background activity on PET imaging. At the time of this analysis, 14/17 patients were alive, median follow-up period of 13 months. All surviving patients are either in remission (undetectable serum PSA levels, *n* = 7) or their disease have stabilized (serum PSA levels and findings on ^68^Ga-PSMA-11 PET/CT remained stable, n = 7).

**Fig. 2:**
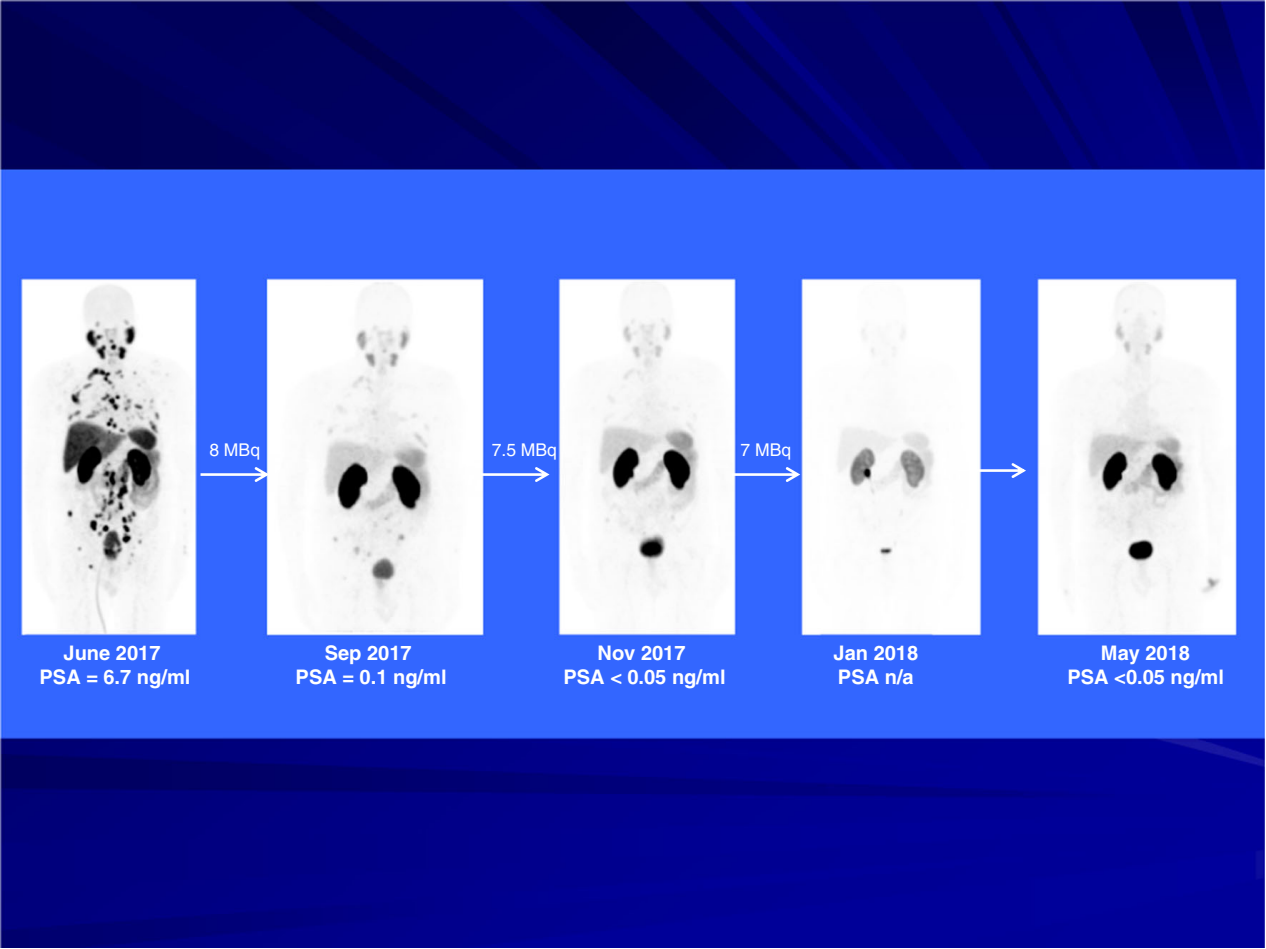
A patient with progressive disease while on androgen deprivation therapy achieved a complete response after three cycles of ^225^Ac-PSMA-617 (patient number 10, Table 1). He remained symptom-free on 11-month follow-up with his serum PSA remaining below detectable level and the follow-up ^68^Ga-PSMA-11 PET/CT scan remaining negative for disease recurrence

**Fig. 3:**
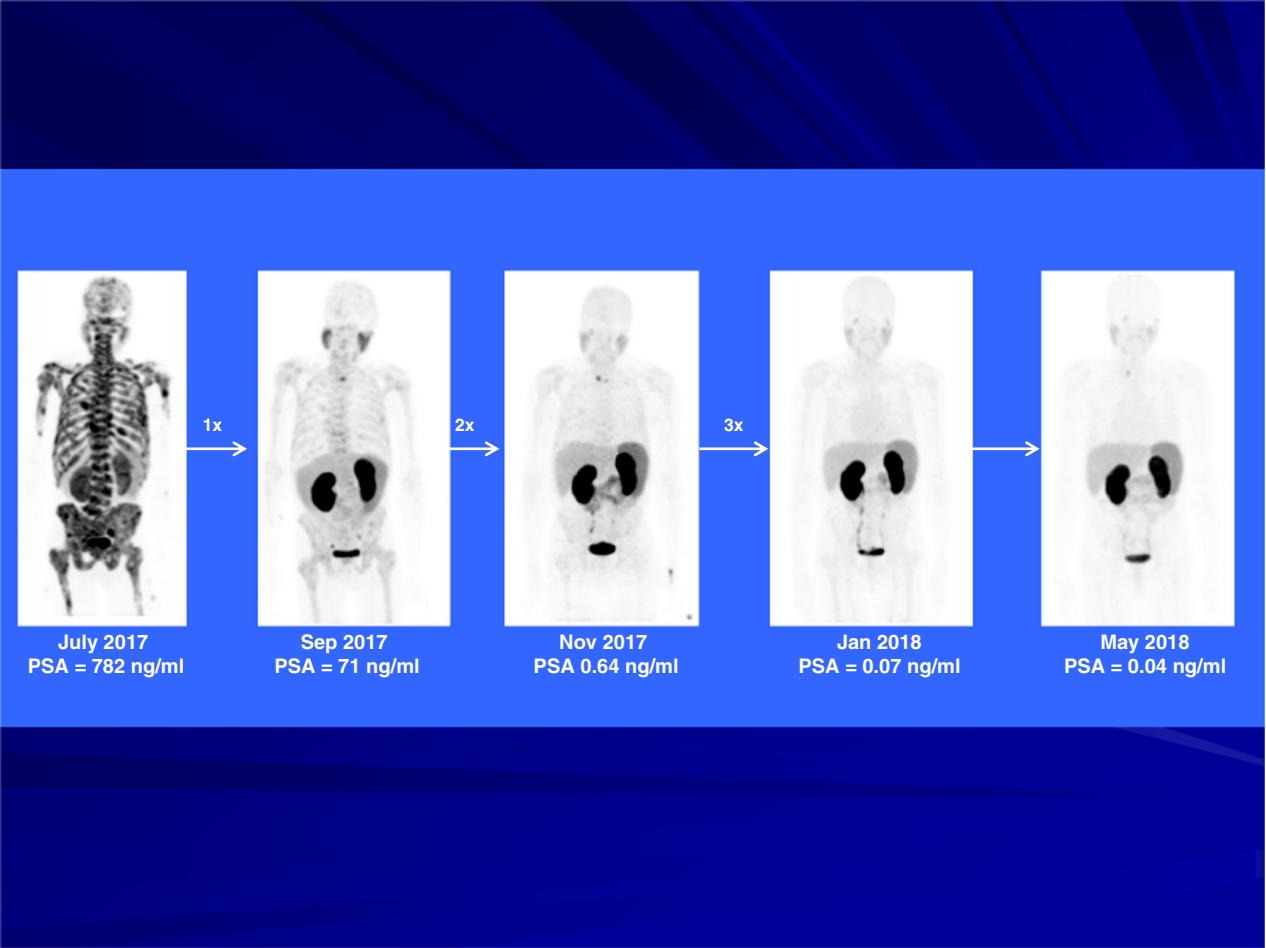
A treatment-naïve patient who presented with extensive bone metastasis at primary diagnosis achieved a complete remission after three cycles of ^225^Ac-PSMA-617 with de-escalating activities of 8/7/6 MBq (patient number 16, Table 1). He also remained symptom-free on 11-month follow-up with his serum PSA remaining below detectable level and the follow-up ^68^Ga-PSMA-11 PET/CT scan remaining negative for disease recurrence

**Fig. 4:**
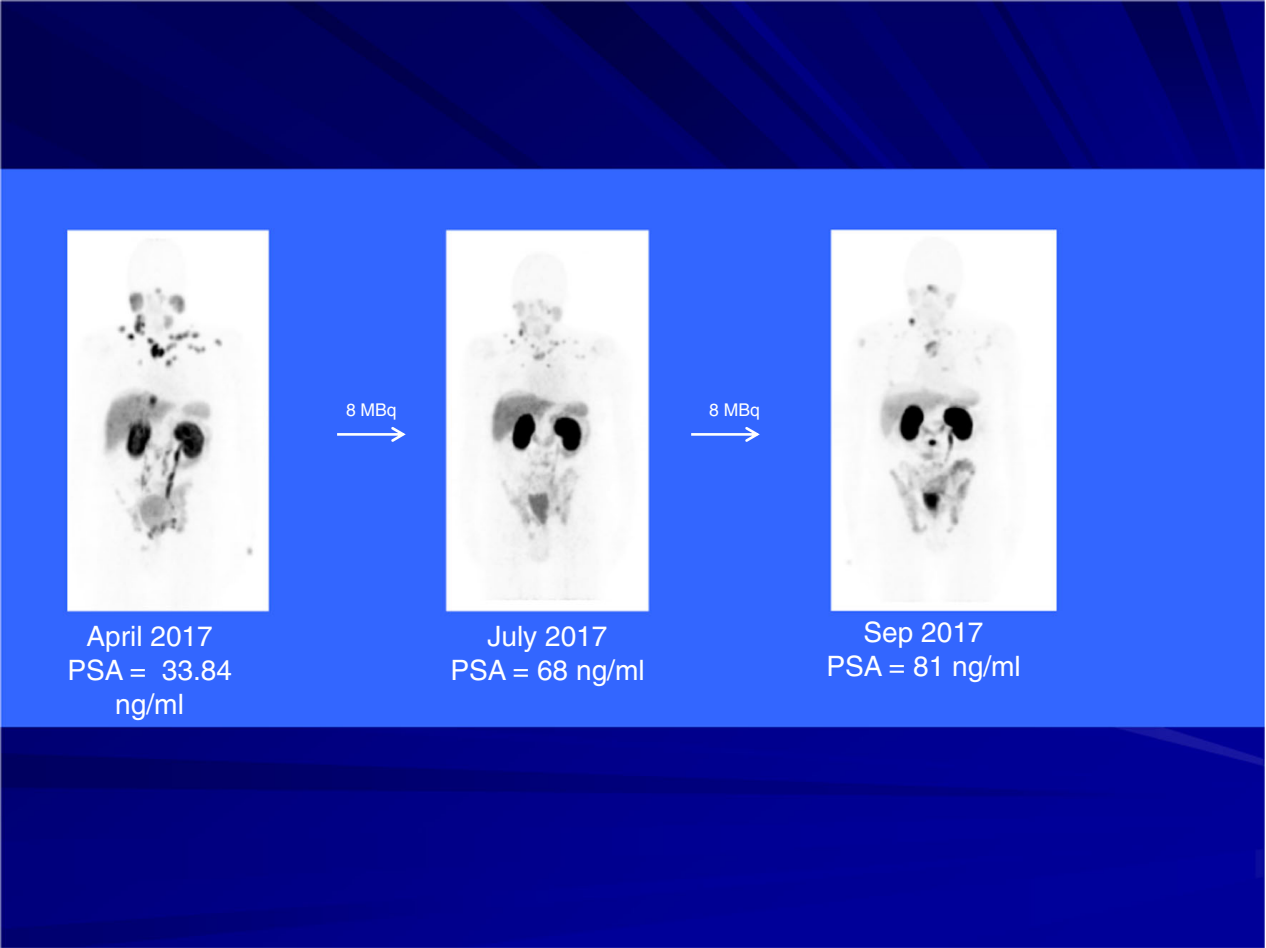
An example of a patient with progressive disease under therapy with ^225^Ac-PSMA-617 after pre-treatment with prostatectomy, external beam radiation and androgen deprivation therapy. The patient decided to switch to chemotherapy and died 4 months after the last cycle of ^225^Ac-PSMA-617 (patient number 1, Table 1)

Figures 2, 3 and 4 show representative images of treated patients. Figure 2 shows a patient with widespread metastatic prostate cancer. His disease progressed while on androgen deprivation therapy with Zoladex. He achieved a complete response after three cycles of ^225^Ac-PSMA-617. He remained symptom-free on 11-month follow-up with his serum PSA remaining below detectable level and the follow-up ^68^Ga-PSMA-11 PET/CT scan remaining negative for disease recurrence. Figure 3 shows a treatment-naïve patient who presented with extensive bone metastasis at primary diagnosis (super scan, alkaline phosphatase = 550 U/L, PSA 782 ng/mL). He achieved a complete remission after three cycles of ^225^Ac-PSMA-617 with de-escalating activities of 8/7/6 MBq. The patient benefitted from significant bone pain palliation already after the first treatment cycle, alkaline phosphatase dropped to 199 U/L after three treatment cycles. He also remained symptom free with undetectable serum PSA and a negative ^68^Ga-PSMA-11 PET/CT at 10-month follow-up evaluation. This patient had grade 2 anemia (hemoglobin = 8.7 g/dL) before initiation of treatment due to extensive bone marrow infiltration. In view of his challenging clinical situation he was offered treatment with ^225^Ac-PSMA-617. His hemoglobin level dropped further to 6.6 g/dL (grade 3 toxicity) after the first cycle of treatment. He had transfusion support at this time and hemoglobin level rose to 9.2 g/dL at the time of third cycle of treatment. His hemoglobin level was 10.3 g/dL at 10 months follow-up without any need for transfusion support. There was no associated grade 1 thrombocytopenia and leucopenia in this patient.

An example of a patient with progressive disease under therapy with ^225^Ac-PSMA-617 is shown in Fig. 4. After pre-treatment with prostatectomy, external beam radiation and androgen deprivation therapy, this patient presented with multiple metastatic lesions and a PSA of 34 ng/mL. While existing lesions showed response to treatment with ^225^Ac-PSMA-617, new lesions developed and his serum PSA level rose to 81 ng/mL. The patient decided to switch to chemotherapy and died 4 months after the last cycle of ^225^Ac-PSMA-617.

### Toxicity

All patients tolerated ^225^Ac-PSMA-617 well. No acute toxicity of the treatment agent was seen in any patient. All patients completed scheduled cycles of treatment, and no patients discontinued treatment due to side effects. All patients experienced grade 1-2 xerostomia. No severe xerostomia (grade 3) was seen in any patient, and no patient required discontinuation of treatment due to dry mouth. One patient with diffuse bone marrow metastases (super scan pattern) had a grade 2 anemia before initiation of therapy. His hemoglobin level deteriorated further after the first cycle of treatment and subsequently recovered fully though transfusion support.

Another patient with one functional kidney had grade 3 renal toxicity before initiation of treatment. In view of the very advanced stage of his disease, the patient was offered treatment and achieved a complete radiological and biochemical response after three treatment cycles. His kidney function deteriorated under treatment. His glomerular filtration rate was 10 mL/min/1.73 m^2^ body surface area (grade 4 toxicity) at 3 months post therapy follow-up.

**Fig. 5:**
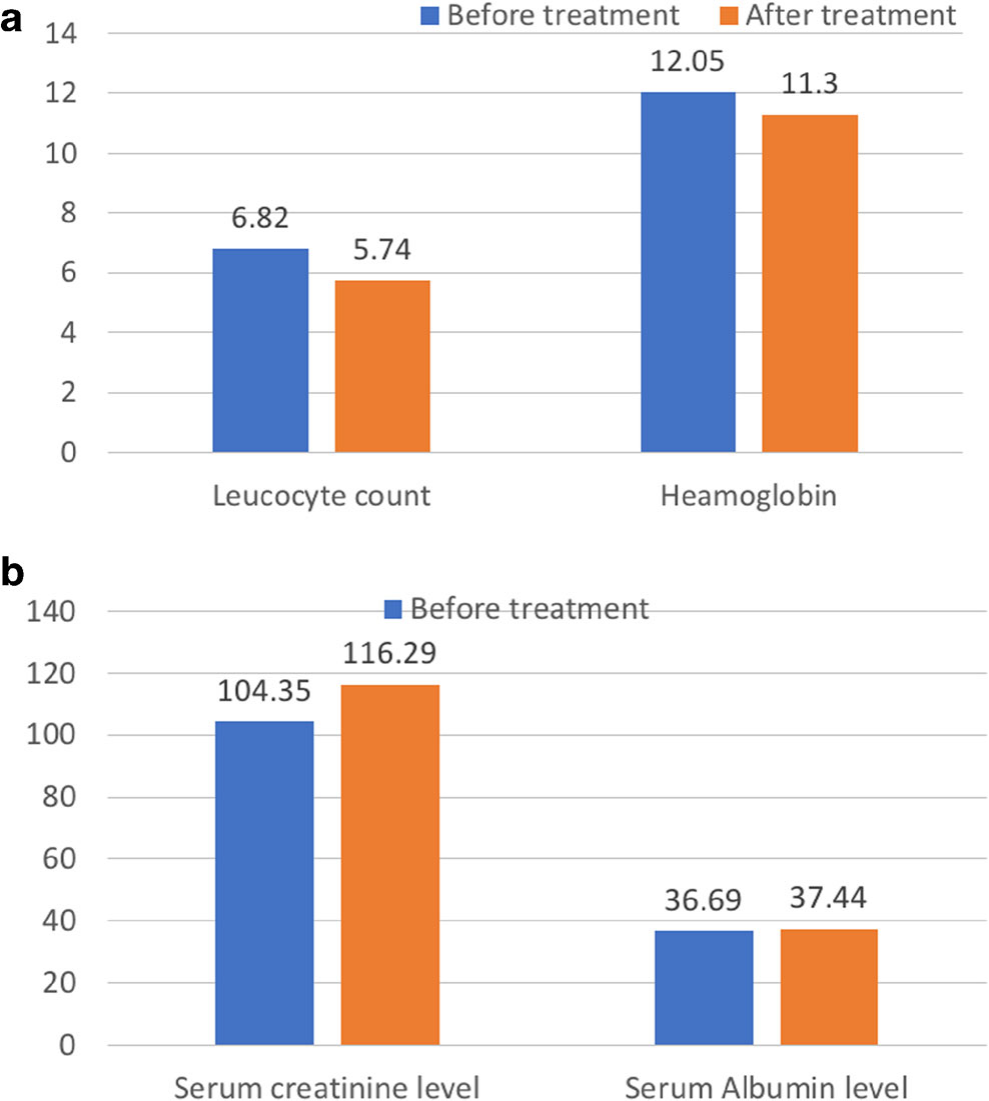
Bar charts comparing results of leucocyte count and hemoglobin (A), as well as serum creatinine and albumin (B) obtained at baseline and at the completion of ^225^Ac-PSMA-617 PRLT. The differences in the pre-treatment and follow-up measurements of leucocyte count *(p* = 0.292), hemoglobin level (*p* = 0.485), serum creatinine level (*p* = 0.683) and serum albumin level (*p* = 0.633) did not reach statistical significance

Figure 5 compares the results of blood tests done to evaluate for adverse events before and after treatment. There was no statistically significant difference between the pre-treatment and follow-up values of leucocyte count (*p* = 0.292), hemoglobin level (*p* = 0.485), platelet count (*p* = 0.216), serum creatinine level (*p* = 0.683), or serum albumin level (*p* = 0.633).

## Discussion

The first report of the remarkable therapeutic efficacy of ^225^Ac-PSMA-617 in patients with late-stage mCRPC presented complete remissions observed in two patients in highly challenging situations [17]. Subsequently, a treatment activity of 100 kBq/kg body weight of ^225^Ac-PSMA-617 per cycle repeated every 8 weeks was determined as a reasonable trade-off between toxicity and biochemical response for advanced-stage patients [16]. In a retrospective analysis of the efficacy of ^225^Ac-PSMA-617 treatment with three cycles of 100 kBq/kg body weight in a group of 40 heavily pre-treated patients with mCRPC, remarkable PSA and a radiological response indicating anti-tumor activity of ^225^Ac-PSMA-617 were shown; at this center most patients had previously received extensive pretreatment with docetaxel (70%), cabazitaxel (17.5%), abiraterone (85%) and enzalutamide (60%), in addition to standard therapies generally including prostatectomy, external beam radiation and androgen deprivation therapy. A swimmer-plot analysis indicated a promising duration of tumor-control, especially taking into account the unfavorable prognostic profile of the selected advanced-stage patients. Xerostomia was the main reason to discontinue therapy or to refuse additional administrations, indicating that further modifications of the treatment regimen concerning side effects are necessary to enhance the therapeutic profile further [15].

In contrast, the clinical challenges for therapy of prostate cancer in South Africa are different. As regular PSA screening is not commonly performed, a majority of patients already presents with widespread, symptomatic disease at primary diagnosis. Novel, second-line androgen deprivation therapies such as enzalutamide or abiraterone are not reimbursed by the public health care system and are not accessible to most patients due to prohibitive high costs. In addition, many patients refuse treatment with chemotherapy or hormonal therapy due to fear of associated side effects. Patients with advanced disease that are not eligible for radio-chemotherapy or decline treatment with chemo- or hormonal therapy represent a high-risk patient population with limited therapeutic options and poor prognosis.

Here we report data from the first 17 consecutive, chemotherapy-naïve patients who presented late with advanced disease and were treated with two to six cycles (median three cycles) of ^225^Ac-PSMA-617 RLT at Steve Biko Academic Hospital in Pretoria, South Africa. For all patients reported here, at least 1-year follow-up is available after initiation of therapy (median 13 months). We are aware that the follow-up period is still limited, in particular in view of monitoring long-term duration of responses and potential long-term toxicity. However, given the high interest in the community in application of ^225^Ac-PSMA-617, we consider it important to share our findings on its remarkable therapeutic efficacy in chemotherapy-naïve patients already at this stage.

Derived from the existing clinical experience on the dosing and toxicity of ^225^Ac-PSMA-617 in heavily pre-treated patients, we further developed the treatment protocol for our patient cohort in several aspects: primarily for reasons of standardization and simplification of procedures, ^225^Ac-PSMA-617 was administered in fixed activities of 8 MBq for the first cycle (corresponding to 100 kBq/kg body weight in a typical patient of 80 kg). Considering the lower number of pre-treatments in our patient group, we expected more frequent and more pronounced responses. Consequently, assessment of radiological response with ^68^Ga-PSMA-11 PET/CT and tumor marker was performed before each subsequent treatment cycle to determine the possibility for de-escalation of administered activities to minimize toxicity, in particular toxicity to salivary glands. In case of favorable response (PSA decline >50% and radiological response), activities of ^225^Ac-PSMA-617 were decreased to doses between 7 MBq and 4 MBq. In three cases, treatment could even be discontinued after two treatment cycles due to excellent response. In one patient who failed to show demonstrable response to the initial two cycles of treatment, activity was escalated to 13 MBq in the third cycle due to clinical need (patient number 3, Table 1). He subsequently showed a decline in serum PSA and a corresponding reduction in tracer uptake in metastatic bone lesions on ^68^Ga-PSMA-11 PET/CT scan. Administered activity was subsequently de-escalated, titrated against residual disease.

Data were analyzed for response after the first and subsequent therapy cycles. Seventy-one percent (12/17) of the patients already responded to the first cycle of RLT with a PSA decline ≥80%. At the end of treatment, 82% (14/17) of patients had a PSA decline of ≥90%. Follow-up ^68^Ga-PSMA-11 PET/CT performed for functional response assessment showed complete lesion resolution in 11 patients (65%), seven of whom also had undetectable serum PSA (<0.1 ng/mL, 41%). As expected from the lower number of pre-treatments of our patients in comparison to the group of 40 heavily pre-treated ^225^Ac-PSMA-617 patients reported earlier [15], the response observed in our cohort are significantly more pronounced with respect to PSA decline and fraction of patients experiencing complete responses.

These interim results also show a favorable hematological and renal toxicity profile of ^225^Ac-PSMA-617 RLT. No significant changes were observed in leucocyte, hemoglobin values pre- and post-therapy (Fig. 5). A patient with a background renal impairment (grade 3 renal failure) developed a grade 4 renal toxicity. Glomerular filtration rate dropped and the serum creatinine level rose after three cycles of ^225^Ac-PSMA-617 therapy (grade 4 toxicity). He was, however, in remission after completing the three cycles of ^225^Ac-PSMA-617 with serum PSA level below the detectable limits at 3 months post completion of treatment. All other patients did not show any signs of acute renal toxicity. However, it is well known that kidney damage following radionuclide therapy can manifest in a delayed manner and onset of radiation-induced renal dysfunction may appear years later and might be missed with our short follow-up period. However, considering an estimated kidney dose of 0.7 Sv_RBE5_/MBq of ^225^Ac-PSMA-617 [21], a cumulative activity of 24 MBq, corresponding to an equivalent dose of 16.8 Sv_RBE5_, still keeps a safety margin to the generally accepted tolerable cumulative equivalent dose of 27 Sv_RBE5_ for kidneys.

The commonest therapy-related adverse effect in our treatment cohort was xerostomia. We found grade 1/2 xerostomia in all patients included in this study. This side effect was tolerable in all patients and did not lead to treatment discontinuation. We de-escalated administered activity in patients who had an excellent initial response to therapy. We started treatment with 8 MBq and de-escalated to 7 to 4 MBq for subsequent therapy sessions titrated against residual tumor burden. This perhaps ameliorated the severity of xerostomia seen in our patients compared with a previous study were 10% of patients discontinued treatment due to intolerable xerostomia [15]. Thus, optimization of the dosing regimen while maintaining sufficient therapeutic efficacy might be a promising strategy for minimization of side effects on salivary glands. However, additional approaches for protection of salivary glands should be developed to further enhance the safety profile of this highly effective therapy. A recent study investigating sialendoscopy with dilatation, saline irrigation and steroid injection showed beneficial effects to the salivation function and health-related quality of life in 11 patients under ^225^Ac-PSMA-617-RLT [22].

The early, but encouraging data presented here indicate that PSMA-based radioligand therapy (RLT) approach could be an attractive therapy option for advanced metastatic prostate cancer patients who are not suitable candidates for conventional therapies with chemotherapy, especially due to toxicities associated with chemotherapeutic agents [9]. There are currently no validated clinical decision tools to aid optimal treatment selection in men with mCRPC [10]. Perhaps a new paradigm could emerge in prostate cancer by moving ^225^Ac-PSMA-617 RLT up the line. When we started our treatments, the evidence levels and development status for ^177^Lu-PSMA-617 and ^225^Ac-PSMA-617 were equal. However, ^177^Lu-PSMA-617 recently finished phase-2 (also with promising results) [23] and is now evaluated in phase-3. Further work will be necessary to elaborate on which patient collective can benefit the most from one particular radio-labeled therapy. Among our 13 castration-resistant patients that had exploited pre-treatment with androgen deprivation therapy, as well as surgery and/or radiotherapy if applicable, 69% (9/13) achieved a PSA decline ≥90% at the end of treatment, including seven patients that achieved complete responses. Given the potential impact that ^225^Ac-PSMA-617 RLT could have on chemotherapy-naïve patients who presented late with advanced and aggressive disease, a prospective clinical trial is required to define the place of targeted alpha therapy with ^225^Ac-PSMA-617 in the management of metastatic prostate carcinoma and its relative efficacy compared with the currently approved therapy modalities.

Factors predictive of response to radioligand therapy have been described for ^177^Lu-PSMA-617 in patients with mCRPC [24]. The modest number of patients reported in this preliminary study, however, preclude a thorough analysis of predictors of response to therapy. We observed, though, that patients who were treatment-naive tend to respond better to ^225^Ac-PSMA-617 RLT compared with patients who were previously treated. For example, all treatment-naïve patients (patients number 8, 9, 15 and 16) achieved >90% decline in their serum PSA after three or four cycles of treatment. In contrary, patients whose disease progressed after two or more previous treatments (for example, patients number 1, 2 and 3) had a less favorable PSA decline following treatment (Table 1, Fig. 1b). The factors predictive of response to ^225^Ac-PSMA-617 RLT in metastatic prostate cancer should be one of the goals of future prospective trials.

In the past, production of ^225^Actinium has relied on radiochemical extraction from ^229^Thorium sources available at JRC Karlsruhe in Germany, Oak Ridge National Laboratory, USA and IPPE in Obninsk, Russia. The current supply from this production route amounts to approximately 68 GBq per year [25]. Additional supply has recently become available from spallation of ^232^Thorium with high energy protons [26]. Considering a typical dose of 8 MBq ^225^Ac-PSMA-617 per treatment cycle, several thousand patient doses can thus be provided per year, allowing the further development of the therapy in large scale prospective clinical trials. Ultimately, further accelerator driven technologies, in particular through proton irradiation of ^226^Radium in medium energy cyclotrons [27], need to be implemented on industrial scale to meet the expected high demand for ^225^Actinium for widespread application of targeted alpha therapy of prostate cancer.

## Conclusion

Remission could be achieved with ^225^Ac-PSMA-617 RLT in chemotherapy-naïve patients with advanced metastatic prostate carcinoma. Remarkable therapeutic efficacy could be achieved with reduced toxicity to salivary glands due to a strategy of de-escalation of administered activities in second and third treatment cycles. This necessitates further exploration for informing clinical practice and clinical trial design.
